# IgG, IgM and IgA antibodies against the novel polyprotein in active tuberculosis

**DOI:** 10.1186/1471-2334-14-336

**Published:** 2014-06-17

**Authors:** Xiaoyan Feng, Xiqin Yang, Bingshui Xiu, Shuang Qie, Zhenhua Dai, Kun Chen, Ping Zhao, Li Zhang, Russell A Nicholson, Guohua Wang, Xiaoguo Song, Heqiu Zhang

**Affiliations:** 1 Department of Bio-diagnosis, Beijing Institute of Basic Medical Sciences, Beijing 100850, China; 2 Chaoyang District Centre for Disease Control and Prevention, Beijing 100029, China; 3 Tianjin Haihe Hospital, Tianjin 300350, China; 4 Department of Biological Sciences, Simon Fraser University, Burnaby, B.C V5A 1S6, Canada

**Keywords:** Tuberculosis, Serodiagnosis, Polyprotein

## Abstract

**Background:**

The present study was aimed to evaluate whether IgG, IgM and IgA antibodies levels detected against a novel *Mycobacterium tuberculosis* polyprotein 38 F-64 F (with 38 F being the abbreviation for 38kD-ESAT6-CFP10 and 64 F for Mtb8.4-MPT64-TB16.3-Mtb8) are suitable for diagnosing active tuberculosis, and for monitoring the efficacy of chemotherapy on TB patients.

**Methods:**

In this study, a total of 371 active TB patients without treatment were selected and categorized into S+/C+ group (n = 143), S-/C+ group (n = 106) or S-/C- group (n = 122). A series of serum samples were collected from 82 active TB patients who had undergone anti-TB chemotherapy for 0–6 months at one month interval. Humoral responses (IgG, IgM and IgA) were determined for the novel *Mycobacterium tuberculosis* polyprotein using indirect ELISA methods in all of serum samples.

**Results:**

For S+/C+, S-/C+ and S-/C- active tuberculosis patients before anti-TB chemotherapy, the sensitivities of tests based on IgG were 65.7%, 46.2% and 52.5% respectively; the sensitivities based on IgM were 21.7%, 24.5% and 18.9%; and the sensitivities based on IgA were 25.2%, 17.9% and 23.8%. By combination of three isotypes, for all active tuberculosis patients, the test sensitivity increased to 70.4% with the specificity being 91.5%. After anti-TB chemotherapy, there were no significant differences between groups with different courses of anti-TB chemotherapy.

**Conclusions:**

The novel *Mycobacterium tuberculosis* polyprotein 38 F-64 F represents potential antigen suitable for measuring IgG, IgM and IgA antibodies. However, the serodiagnostic test based on the 38 F-64 F polyprotein appears unsuitable for monitoring the efficacy of chemotherapy.

## Background

Tuberculosis (TB) remains the leading single microbial illness globally, with one-third of the world’s population infected with *Mycobacterium tuberculosis* (*M. tuberculosis*, Mtb) complex. In 2009, there were over 9.4 million new cases and 1.7 million deaths from *M. tuberculosis*[1]. Over 90% of the worldwide burden of tuberculosis is in low-income and middle-income countries where the diagnosis of tuberculosis still relies heavily on sputum smear microscopy and chest radiology. There is a great need for rapid point-of-care tests that can be readily used at all levels of the health system and in the community [2,3].

The identification of the bacillus by microscopic examination of sputum smear or by culture, however, presents certain limitations. Around 30-50% of TB patients are negative in the microscopy examination, and culture requires a long time for the growth of *M. tuberculosis*, which maybe lead to delay diagnosis [4,5]. The tuberculin skin test has long been used for the diagnosis of TB. While this test is the recommended diagnosis test for latent TB infection, it requires standardized application and interpretation, and a positive result depends on an adequate immune response [6]. The usefulness of the IFN-γ-release assays in the diagnosis of active TB remains questionable [7]. Nucleic acid amplification tests, for example the Xpert MTB/RIF assay, are the most promising development in tuberculosis diagnostics in the USA and Europe [8]. However, uses of such tests are restrictive because this assay requires dedicated and expensive equipment. The serological test based on the detection of circulating antibodies against *M. tuberculosis*-specific antigens could represent a useful complement to microscopic examination for screening active tuberculosis [9]. This method is quite attractive because of its easy application, low cost of testing many serum samples in parallel and relatively low invasiveness.

A comprehensive insight to immunoprofiling of antigen specific responses is critical for TB diagnosis and therapeutic monitoring. Currently, gold standard methods to diagnosis TB and monitor treatment response include sputum smear microscopy and culture conversion after 2 months of TB treatment. But, for patients in whom such sputum sample is not available, alternative serological tests are needed. Some results showed that combined use of different antibody isotypes allow an increased accuracy in diagnostic of tuberculosis [10], and the levels of antibody against to some antigens decreased together with treatment [11]. On the contrary, some results showed that combination of IgG with IgA and/or IgM does not improve its sensitivity, and the levels of antibody against to other antigens were not associated with anti-TB treatment [12].

In our previous study, two novel *M. tuberculosis* polyproteins, 38 F (38kD-ESAT6-CFP10) and 64 F (Mtb8.4-MPT64-TB16.3-Mtb8), were expressed as antigens with multiepitopes, and evaluated for serodiagnosis of TB. The novel 38 F-64 F indirect ELISA assay based on measuring IgG antibody has potential to achieve higher sensitivity and specificity, and the ROC analysis indicated that the novel 38 F-64 F indirect ELISA assay had a better overall diagnostic performance [13].

The goals of the present study were to evaluate serum levels of all of three isotype antibodies, IgG, IgM and IgA, specific to 38 F-64 F, in patients with active TB and in BCG-vaccinated healthy individuals, and to assess whether determination of 38 F-64 F specific antibody responses could be useful for monitoring the efficacy of chemotherapy.

## Methods

### Ethics statement

The study was approved by the ethics committee of the Beijing Chaoyang District Centre for Disease Control and Prevention, and Tianjin Haihe Hospital. Written informed consent was obtained from all participants.

### Study population

Sera were obtained from patients with active TB and from normal individuals, as described below. Diagnosis of active TB was established by the presence of clinical symptoms of TB, by chest radiography, and by symptomatic improvement after chemotherapy. Sera were stored at −70°C until start of testing.

A total of 371 active TB patients at their first visit to the outpatient clinic without treatment were selected and categorized into three groups, i.e.

S+/C+: Smear-positive and culture-positive group (n = 143), with 82 males and 61 females and the age range from 19 to 67, with a median of 42.

S-/C+: Smear-negative and culture-positive group (n = 106), with 55 males and 51 females and the age range from 22 to 70, with a median of 46.

S-/C-: Smear-negative and culture-negative group (n = 122), with 65 males and 57 females and the age range from 19 to 78, with a median of 40.

Sputum samples were processed using standard NALC-NaOH method and smears were examined after Ziehl–Neelsen staining. Processed samples were inoculated in MGIT (Mycobacterial growth indicator tube) 960 non-radiometric automated isolation system (BD, USA) in accordance with the standard procedure.

To assess whether determination of 38 F-64 F specific antibody responses could be useful for monitoring the efficacy of chemotherapy, a series of serum samples were obtained from 82 active TB patients, which including 30 IgG positive patients, 28 IgA positive patients and 24 IgM positive patients at their first visit to the outpatient clinic. The diagnosis of active TB in these patients was based on sputum smear positive and/or sputum culture positive and/or X-ray clinical findings. All patients had received combination antituberculosis chemotherapy with isoniazid and rifampin and pyrazinamide and ethambutol/streptomycin for 2 months followed by admission isoniazid and rifampin for 4 months. All patients were HIV seronegative and there were no other disease states accompanying the TB. A series of serum samples were collected at one month interval, and divided into seven groups, i.e., 0 M, 1 M, 2 M, 3 M, 4 M, 5 M and 6 M.

Ninety-four BCG-vaccinated healthy blood donors were included. Their age ranged from 20 to 55, with a median of 38. The healthy subjects had normal findings on chest radiogram and no history of close contract with TB patients and no family history of tuberculosis. These subjects were apparently normal without HIV infection and other diseases that might be confused with TB such as pneumonia, fungal infections, lung cancer, etc.

### ELISA

Microplates were coated with individual antigens at 5 μg/ml (3 μg/ml 38 F and 2 μg/ml 64 F) in coating buffer (0.05 M carbonate/bicarbonate, pH 9.6) and stored at 4°C overnight. The plates were washed three times with phosphate-buffered saline (PBS) containing 0.05% Tween 20 (PBST). Two hundred microliters of PBST containing 1% bovine serum albumin was added to each well, and the plates were sealed and incubated at 37°C for 1 h. The plates were washed three times. One hundred microliters of serum diluted 1:10 in PBST containing 1% BSA was added to each antigen-coated well. The plates were sealed and incubated at 37°C for 30 min and then washed three times. One hundred microliters of horseradish peroxidase-conjugated anti-human IgG antibody (Sigma, USA), anti-human IgM antibody (Bethyl, USA) or anti-human IgA antibody (Bethyl, USA) was added to each well respectively, and the plates were sealed and incubated at 37°C for another 30 min. Again, the plates were washed three times. The bound enzyme was detected by freshly-prepared tetramethyl benzidine (TMB) substrate. After 20 min incubation in room temperature, the stop solution (0.1 N sulfuric acid) was added and the optical density was determined at 450 nm using an automatic microplate reader (Bio-Rad, USA).

### Statistical analysis

Data processing was performed using GraphPad Prism 4.0 (GraphPad Software Inc., San Diego, CA) and SPSS16.0 software package (SPSS Inc., Chicago, IL). The diagnostic value of the novel 38 F-64 F indirect ELISA assay was evaluated by the receiver operating characteristic (ROC) curve performed with the data from patients with active TB and 94 BCG-vaccinated healthy blood donors. The cutoff value of 38 F-64 F IgG, IgM and IgA were chosen according to the ROC analysis. Comparisons between treatment groups were done by the Mann Whitney test.

## Results

### IgG, IgM and IgA antibody responses against 38 F-64 F antigens in active TB without treatment

**Figure 1:**
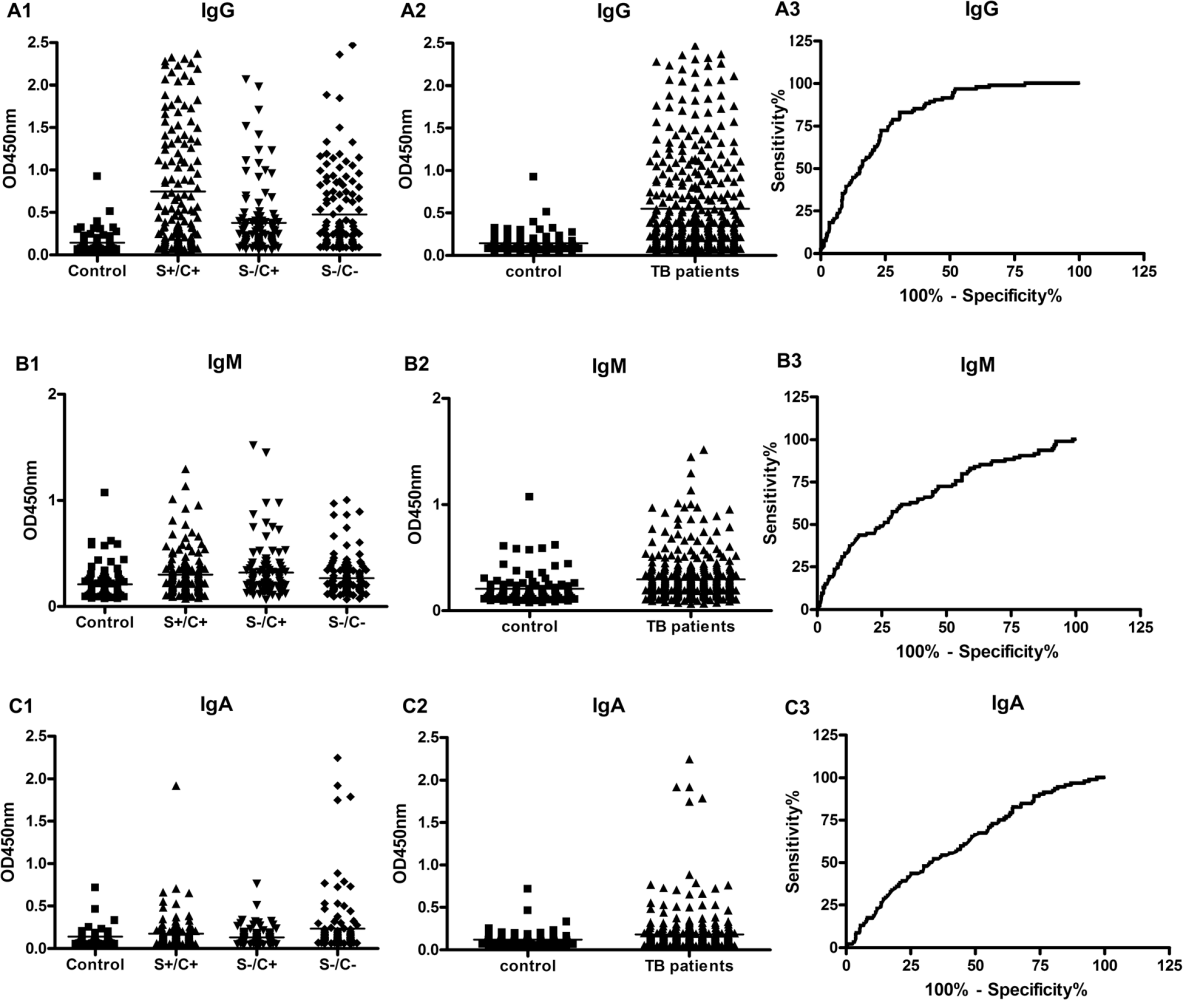
**The serodiagnostic performance of the novel 38 F**-**64 F indirect ELISA assay in active TB patients at their first visit to the outpatient clinic without treatment.** Levels of serum IgG, IgM and IgA against the novel 38 F-64 F polyprotein in S+/C+, S-/C + and S-/C- TB patients **(A1, B1 and C1)** and in total TB patient **(A2, B2 and C2)** were presented as scatter grams respectively. Each bar represents the mean OD value. The ROC curves of the novel 38 F-64 F indirect ELISA assay based on measuring IgG **(A3)**, IgM **(B3)** and IgA **(C3)** were shown and the areas under the curve (AUC) for IgG, IgM and IgA were 0.81, 0.68 and 0.62 respectively.

In our previous study, the novel 38 F-64 F antigens were confirmed to have potential for higher sensitivity and specificity based on measuring IgG antibody [13]. Considering the heterogeneity of the humoral response, in this study, serum levels of all of three isotype antibodies, IgG, IgM and IgA, specific to 38 F-64 F were evaluated in active TB patients at their first visit to the outpatient clinic without treatment. The data are shown in Table 1 and presented as scattergrams in Figure 1.

**Table 1:** **ELISA results of the 38 F**-**64 F antigens in the serodiagnosis of active pulmonary TB**

**Group**	**S+/C + (n = 143)**			**S-/C + (n = 106)**			**S-/C- (n = 122)**		
	**IgG**	**IgM**	**IgA**	**IgG**	**IgM**	**IgA**	**IgG**	**IgM**	**IgA**
# Pos. (%)	94 (65.7)	31 (21.7)	36 (25.2)	49 (46.2)	26 (24.5)	19 (17.9)	64 (52.5)	23 (18.9)	29 (23.8)
# Pos. for one isotype of antibody	53	4	8	29	11	5	39	9	11
# Combin. Pos. (%)	108 (75.5)			68 (64.2)			85 (69.7)		

S+/C+: smear-positive and culture-positive active pulmonary TB patients; S-/C+: smear-negative and culture-positive active pulmonary TB patients; S-/C-: smear-negative and culture-negative active pulmonary TB patients; # Pos. (%): number of positive sera (percentage of positive sera); # Combin. Pos. (%): number of combination positive sera for any of three isotype antibodies (percentage of combination positive sera).

Before anti-TB chemotherapy, the sensitivities of the tests based on IgG were 65.7% (95% CI, 57.3% to 73.5%), 46.2% (95% CI, 36.5% to 56.2%) and 52.5% (95% CI, 43.2% to 61.6%) in S+/C+, S-/C + and S-/C- active tuberculosis patients respectively; the sensitivities of the tests based on IgM were 21.7% (95% CI, 15.2% to 29.3%), 24.5% (95% CI, 16.7% to 33.8%) and 18.9% (95% CI, 12.3% to 26.9%) respectively; and the sensitivities of the tests based on IgA were 25.2% (95% CI, 18.9% to 33.9%), 17.9% (95% CI, 11.2% to 26.6%) and 23.8% (95% CI, 16.5% to 32.3%) respectively. In S+/C+ group, 53, 4 and 8 sera were IgG-, IgM- and IgA-positive only, respectively, and the number of combination positive sera was 108, which including anyone, any two and all of three isotype of antibodies positive sera. By combination of all the three isotype an increased sensitivity from 65.7% to 75.5% was obtained. In S-/C+ group and S-/C- group, the sensitivity increased from 46.2% to 64.2% and from 52.5% to 69.7% by combination of all the three isotype respectively.

To evaluate the specificity, all the three isotype antibodies responses were examined in 94 BCG-vaccinated healthy blood donors. The specificity of the tests based on IgG, IgM and IgA were 94.7% (95% CI, 88.0% to 98.3%), 92.6% (95% CI, 85.3% to 97.9%) and 95.7% (95% CI, 89.5% to 98.8%) respectively (Figure 1). By combination of three isotypes, the specificity of the tests decreased slightly to 91.5% (95% CI, 87.0% to 94.8%). These results are consistent with the results of Uma Devi et al. [14]. In that article, when IgG was taken individually, the specificity was 100%, when IgG + IgA were taken, the specificity reduced to 96%, and when IgG + IgA + IgM were taken, the specificity reduced to 90%.The serodiagnosis performance of the novel 38 F-64 F polyprotein on measuring IgG, IgM and IgA antibody was analyzed with all TB patients and healthy controls (Figure 1). The area under the ROC curve (AUC) of the novel 38 F-64 F indirect ELISA assay on measuring IgG, IgM and IgA antibody were 0.81 (95% CI, 0.75 to 0.85), 0.68 (95% CI, 0.61 to 0.74) and 0.62 (95% CI, 0.56 to 0.69) respectively. The ROC analysis indicated that the novel 38 F-64 F polyprotein had a better diagnostic performance on measuring IgG than IgM and IgA.

### Integration of serodiagnostic test and bacteriology examination to screen for active TB

**Figure 2:**
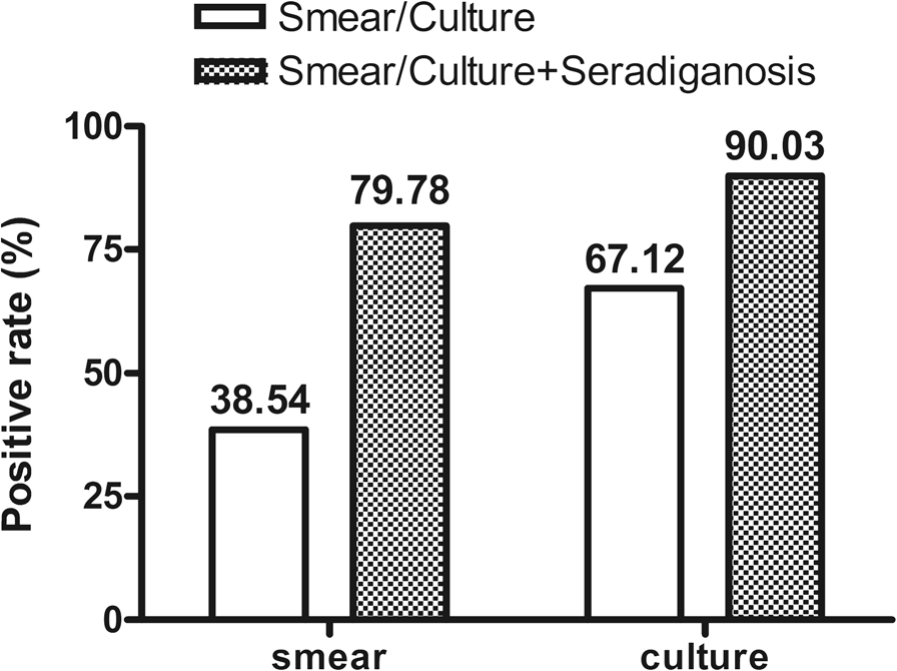
**The positive rates of sputum smear**/**culture with or without integration of serodiagnostic test.** The integration of serodiagnostic test with sputum smear or sputum culture increased the positive rates from 38.54% to 79.78% or from 67.12% to 90.03%, respectively.

By combination of all of three isotypes (IgG or IgM or IgA), a sensitivity of 70.4% (261/371) was obtained with a specificity of 91.5% (86/94) in all of three groups of TB patients without treatment. Moreover, the positive rates of antibodies specific to 38 F-64 F for smear-positive, smear-negative, culture-positive or culture-negative patients were not significantly different. In smear-negative or culture-negative patients, the positive rates of antibodies specific to 38 F-64 F were 67.1% and 69.7%, slightly lower than the positive rates for smear-positive (75.5%) or culture-positive (70.68%) patients (Table 2).The diagnostic sensitivities of sputum smear and sputum culture in all of three group TB patients were 38.5% and 67.1% respectively. The serodiagnostic test using the novel 38 F-64 F polyprotein significantly improved the detection rate for the culture-negative or smear-negative active TB patients. By integration with the serodiagnostic test, the diagnostic sensitivities to active TB for sputum smear or sputum culture increased from 38.5% to 79.8% and from 67.1% to 90.0%, respectively (Figure 2).

**Table 2:** **Serodiagnosis results of the 38 F**-**64 F antigens in the active pulmonary TB patients with different sputum bacteriology results**

**Group**	**S + (n = 143)**	**S- (n = 228)**	**C + (n = 249)**	**C- (n = 122)**
# Pos. (%)	108 (75.5)	153 (67.1)	176 (70.7)	85 (69.7)

S+: smear-positive active pulmonary TB patients; S-: smear-negative active pulmonary TB patients; C+: culture-positive active pulmonary TB patients; C-: culture-negative active pulmonary TB patients; # Pos. (%): number of positive sera (percentage of positive sera).

### Time-course changes in IgG, IgM and IgA antibody titres after initiation of anti-TB chemotherapy

**Figure 3:**
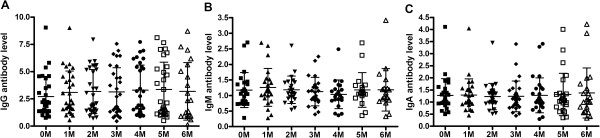
**Levels of serum IgG (A)**, **IgM (B) and IgA (C) against the novel 38 F**-**64 F polyprotein in active TB patients with different course of chemotherapy.** 0 M = patients at their first visit to the outpatient clinic, 1 M-6 M = patients received anti-TB chemotherapy for 1–6 months. The mean antibody level and the standard deviation of each group were shown. There was no significant difference between the patients at their first visit to the outpatient clinic without anti-TB chemotherapy and those receiving anti-TB chemotherapy for 1–6 months. All of the p values between each of two groups were greater than 0.05.

**Figure 4:**
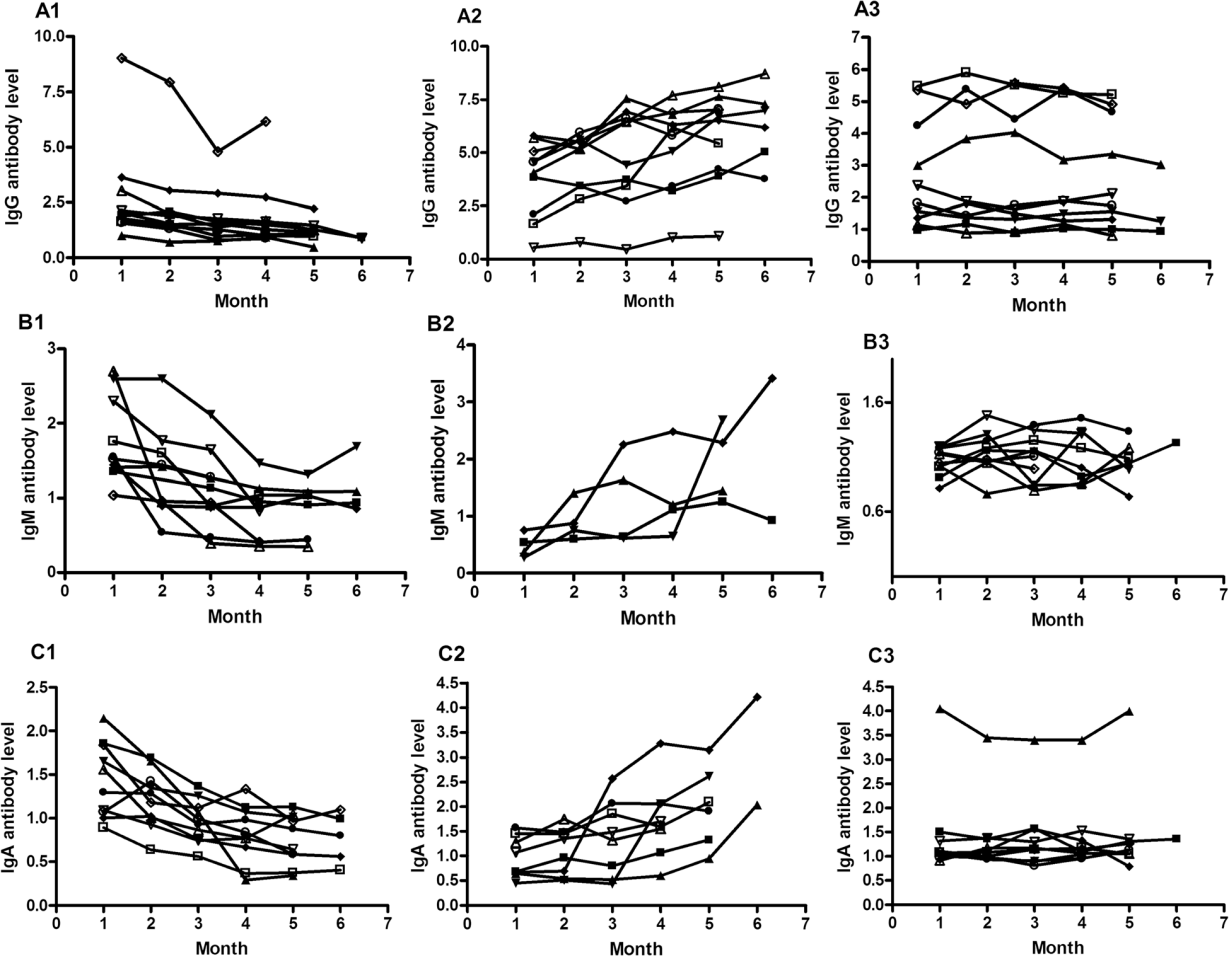
**Changes in IgG (A1, A2 and A3), IgM (B1, B2 and B3) and IgA (C1, C2 and C3) antibody levels against the novel 38 F**-**64 F polyprotein after initiation of anti**-**TB chemotherapy.** After initiation of anti-TB chemotherapy, patients showed three different IgG, IgM and IgA antibody responses patterns, i.e., decrease, increase and maintaining at stable level.

To assess whether determination of 38 F-64 F specific antibody responses could be useful for monitoring the efficacy of chemotherapy, a series of serum samples were obtained from active TB patients who had undergone anti-TB chemotherapy for 0–6 months at one month interval and the serum levels of all of three isotype antibodies, i.e., IgG, IgM and IgA, specific to 38 F-64 F, were determined. The results are presented as scatter grams in Figure 3.In this study, the mean levels of IgG, IgM and IgA antibodies against 38 F-64 F polyprotein in sera of active TB patients with different courses of anti-TB chemotherapy were shown to be no significant difference when compared with results from the same patients at their first visit to the outpatient clinic without anti-TB chemotherapy (P > 0.05). The mean antibody levels and the standard deviation of each groups are shown in Figure 3. After anti-TB chemotherapy was initiated, the IgG, IgM and IgA antibody responses were heterogeneous and differed considerably from patient to patient, although the response patterns can be categorized into three types. The antibody levels against 38 F-64 F in some patients increased with the course of anti-TB chemotherapy. In contrast, in some patients, the antibody levels against 38 F-64 F decreased with the course of anti-TB chemotherapy. The third type of response pattern indicated that the antibody levels against 38 F-64 F were more stable with slightly fluctuations. Some corresponding examples are shown in Figure 4.

## Discussion

An effective *in vitro* diagnostic for TB based on serological methods could be an attractive area of exploration because immunoassays have advantages of simplicity, rapidity and low cost, and have possibility to find the cases missed by standard sputum smear microscopy [2,3,15]. Nevertheless, considerable progress has been made in the identification of many new serological antigens in recently years, and the sensitivity and specificity of serological tests are improving. It is therefore generally accepted that it will be advantageous to include several antigens in a future serodiagnostic assay. In our previous study, the novel polyprotein 38 F-64 F including seven Mtb antigens had been found to achieve higher sensitivity and specificity based on measuring IgG antibody [13]. The knowledge of the humoral immune responses at various stages of TB infection and disease could help to elucidate the complex interaction between host and pathogen [16-18]. A comprehensive insight to immunoprofiling of antigen specific responses is critical not only for the understanding of disease pathogenesis, but also for development of diagnostic tests. In view of the heterogeneity of the humoral response, in this study, serum levels of three 38 F-64 F-specific antibody isotypes, IgG, IgM and IgA, were evaluated in active TB patients at their first visit to the outpatient clinic without treatment and those who had undergone anti-TB treatment.

IgG antibody level is higher in most advanced and extensive forms of the disease. Patients with active TB usually exhibited strong IgG responses but poor IgM and IgA responses [19]. While the function of anti-*M. tuberculosis* antibodies in providing protective immunity is still under investigation, it has been proposed that they may be utilized as a diagnostic marker of active disease [18,19]. Previous studies analyzing IgG antibodies showed that anti-*M. tuberculosis* IgG antibodies increased in patients with active disease [20-22]. In our results, IgG antibody level is higher in the most active TB patients with or without the anti-TB chemotherapy than that in health control, and the positive rate of IgG was highest among the three isotypes, indicating that the IgG antibody was the most extensive antibody isotype.

Usually, IgM antibodies appear first and are produced in large quantities in response to any antigen and decline in more advanced phases. Several authors suggested that IgM antibodies are produced mainly during the early phase of primary TB infection [23,24]. Therefore, the IgM-positive patients were diagnosed at an early stage of the infection process [19,25]. Our results demonstrate that the positive rate of IgM is lowest among the three isotypes. In China, most people visit a doctor only after symptoms appear. Accordingly, at their first visit to the outpatient clinic most TB patients are over the early stage of the infection process. Interestingly, following different stages of the infection process, three types of response patterns of IgM antibody were observed. The antibody levels of IgM against 38 F-64 F in some patients became lower with the course of treatment, but IgM antibody levels in some patients were elevated or were maintained at more stable levels throughout the course of anti-TB chemotherapy. Several authors have observed that IgM antibody production was not associated with any clinical phases and radiological factor [26].

IgA is secreted during the contact of *M. tuberculosis* and/or its antigens with the mucosal surface, which stimulates the release of cytokines [27]. Several authors have described the presence of IgA against the mycobacterial antigens in the serum of TB patients [26,28-31], suggesting that this immunoglobulin isotype is present in both patients with newly acquired infections and those of a longer duration [32]. In the present investigation, the positive rate of IgA was not too high in active TB patients at their first visit to the outpatient clinic and the IgA antibody levels were maintained for a longer duration until the completion of anti-TB chemotherapy.

Secondly, from our results, we found that some sera are IgM- or IgA-positive only. Though most of the active TB patients (70.4%) could be diagnosed by the detection of IgG antibody isotype, few TB patients showing IgM- or IgA-positive only [about 13% (48/371)], were left out, which would lead to delay in treatment and more extensive transmission of TB. Thus, it should detect different types of antibodies simultaneously. And in our results, the test sensitivity was improved for IgG + IgA + IgM without significantly compromising the specificity. These results are consistent with the results of Uma Devi *et al*. [14]. On the other hand, if one were to test all of three antibody isotypes, the expense of diagnosis for TB patient would increase. Therefore, in order to increase the positive rate and decrease the expense of diagnosis, it would be necessary for researchers to develop the total antibody detection method, i.e., double antigen sandwich ELISA.

The other significant result obtained in this study was that the positive antibody responses in smear-negative and/or culture-negative active TB patients were not different significantly with those in smear- and culture-negative active TB patients. This is in uncoincidence with some other studies results showing that smear-positive TB patients generate more proteinaceous antigens than smear-negative TB patients [33,34]. On the other hand, there are a few studies showing that the antibody level does not always correspond to the bacterial load in TB patients [35,36]. Thus, for serodiagnostic methods, there is greater value for combination with, not replacement of, the sputum smear and sputum culture test. The positive rate would increase in paucibacillary TB patients, which are difficult to diagnose by bacteriological tests [12,26]. The findings of Kanaujia GV *et al*. [9] also demonstrated that the effectiveness of the screening strategy was improved by integrating multi-antigen ELISA with microscopic examination of sputum for acid-fast bacilli in areas with high TB prevalence.

In order to assess whether determination of 38 F-64 F specific antibody responses could be useful for monitoring the efficacy of chemotherapy, the levels of three isotype of antibodies were measured in active TB patients with different course of anti-TB chemotherapy. On one hand, the heterogeneity of the antibody response between patients was confirmed by several researchers in previous studies [37-39]. We also observed the antibody response to be heterogeneous in patients with active TB undergoing anti-TB chemotherapies. On the other hand, many investigations concerning the changes in antibody levels showed that antibody levels decrease after initiation of anti-TB chemotherapy [40,41]. Our results did not support such a conclusion. Based on our statistical results, there was no significant difference between groups with different course of anti-TB chemotherapy for all of subjects, and the response patterns could be grouped into three types, i.e. increase, decrease and maintaining at a stable level. Moreover, the antibody against Mtb antigen continued to be present throughout the anti-TB chemotherapy.

A wide spectrum of humoral responses exist in TB patients, depending on different antigens, the expression levels of different antigens, the patient’s immunological background, the disease stage, and/or the type of anti-TB therapy [42,43]. Although higher sensitivity and specificity would be obtained based on measuring antibody against polyprotein, such as 38 F-64 F including seven proteins from Mtb, the regular change in the level of antibody against only one antigen would be covered up. Therefore, the polyprotein may not be suitable for monitoring the efficacy of chemotherapy.

## Conclusions

Based on our present experiments we can make recommendations that may be helpful in subsequent studies on the serological diagnosis of TB. First, all of three 38 F-64 F-specific antibody isotypes, IgG, IgM and IgA, were detected in the sera of TB patients, indicating that the novel 38 F-64 F polyprotein was suitable for diagnosing active TB and it is necessary to develop the double antigen sandwich ELISA to detect total antibodies. Second, the positive rate would increase in the paucibacillary TB patients by serodiagnostic methods. Thus, an improved screening strategy can be created by integrating multi-antigen ELISA with bacteriology tests in areas with high TB prevalence. Third, the serodiagnostic test based on polyprotein antigens does not appear to be useful for monitoring the efficacy of chemotherapeutics or as a criterion to determine whether the patient should continue or terminate anti-TB chemotherapy. Therefore, it will be necessary to evaluate novel predictors of the efficacy of anti-TB drugs based on other immunological markers, such as the level of antigen, cytokine, microRNA, alone or in combination.

## Abbreviations

TB: Tuberculosis; ROC: Receiver operating characteristic; AUC: Area under the ROC curve.

## Competing interests

The authors declared that they have no competing interests.

## Authors’ contributions

The conception and design of the study: XYF, XQY, HQZ; Acquisition of data: XQY, BSX, SQ, ZHD, KC, XGS, GHW; Collection of samples: PZ, L Zhang; Analysis and interpretation of data: XYF, XQY, HQ Zhang; Drafting the article or revising it critically for important intellectual content: XYF, RAN; Final approval of the version to be submitted: XYF, HQZ.

## Pre-publication history

The pre-publication history for this paper can be accessed here:

http://www.biomedcentral.com/1471-2334/14/336/prepub
